# Rapid genome-wide introgression reveals fitness advantage of immigrant genotypes

**DOI:** 10.1101/2025.08.27.672692

**Published:** 2025-08-28

**Authors:** Ben A. Flanagan, Arshad Padhiar, Foen Peng, Saif Quraishi, Andrea J. Roth-Monzón, Fahad Gilani, Lauren Simonse, Meghan F. Maciejewski, Noah Reid, Milan Malinsky, Amanda K. Hund, Daniel I. Bolnick

**Affiliations:** 1 Department of Ecology and Evolutionary Biology, University of Connecticut; 75 North Eagleville Rd, Storrs CT 06269 USA; 2 Current affiliation: Department of Biology, Haverford College; 370 Lancaster Ave, Haverford PA 19041 USA; 3 Current affiliation: Chicago College of Osteopathic Medicine; 555 31^st^ St., Downers Grove IL 60515, USA; 4 Current affiliation: Museum of Vertebrate Zoology, University of California Berkeley; 3040 Valley Life Sciences, Berkeley CA 94720-3140 USA; 5 Current affiliation: School of Integrative Biology, University of Illinois, 505 S. Goodwin Ave, Urbana, IL 61801, USA; 6 Department of Molecular and Cell Biology, University of Connecticut; 75 North Eagleville Rd, Storrs CT 06269 USA; 7 Institute of Ecology and Evolution, University of Bern; Bern, Switzerland; 8 Department of Biology, Carleton College, Northfield, Minnesota, USA.

## Abstract

Evolutionary biology has long recognized the tendency for populations to be locally adapted to their ancestral habitat, resulting in higher resident fitness. However, immigrants can also introduce beneficial alleles. The resulting adaptive introgression is usually inferred retrospectively, rather than as a contemporary process. Here, we document exceptionally rapid ongoing adaptive introgression in a lake population of threespine stickleback (*Gasterosteus aculeatus*). In the first generations after a discrete immigration event, all chromosomes exhibited large increases in immigrant ancestry due to linkage disequilibrium. After a decade, the extent of introgression varied across the genome. The fastest-evolving genes included *Spi1b*, which enables an increased fibrosis defense against a previously common tapeworm, whose prevalence then declined dramatically. This case study highlights the capacity for immigration to supply beneficial alleles that drive rapid genome-wide evolution.

Evolutionary theory leads us to expect local adaptation – the tendency for natives in a particular environment to have higher fitness than immigrants (1). Generations of natural selection retain locally beneficial alleles and remove locally harmful alleles, whereas immigrants carry alleles favored in their native range but untested in their new environment. The resulting selection against immigrant genotypes (2) facilitates population divergence and speciation (3). Yet, immigrants can outperform residents (4). They may import universally beneficial alleles that arose elsewhere in the species range, or may carry alleles pre-adapted to changing local environments. For instance, if parasites gain an edge during coevolution, then resident hosts can be locally maladapted compared to immigrants (5). Once introduced, beneficial immigrant alleles can increase in frequency leading to adaptive introgression (6). Examples of adaptive introgression exist in plant and animal species (7–9), and often entail introgression of alleles at immune genes such as MHC, conferring enhanced resistance to locally adapted parasites (10– 12). However, we know little about the earliest phases of adaptive introgression, which is usually inferred long after the actual event by identifying genomic regions with atypical ancestry (e.g., Neanderthal sequences in modern human genomes (13)). This retrospective inference makes it difficult to determine the exact timing and speed of introgression, and its initial genomic footprint. Here, we use genome sequences spanning two decades to document a contemporary example of adaptive introgression between conspecific populations of threespine stickleback. We reveal very strong selection in the earliest generations of introgression, driving genome-wide increases in immigrant ancestry for all chromosomes. In later generations, introgression began to differ among chromosomes and loci allowing us to identify likely targets of selection, such as a gene conferring immunity to a formerly abundant tapeworm.

The threespine stickleback (*Gasterosteus aculeatus*) is a small fish found in northern coastal habitats. After Pleistocene deglaciation, marine stickleback repeatedly invaded and then adapted to freshwater habitats, including loss of armor plating, and gain of resistance to freshwater parasites (14, 15). As the many isolated lake populations adapted to their respective habitats and communities, they diverged in morphology (16), and immunity (17). For some traits this among-population divergence evolved in parallel, creating replicated trait-environment correlations that strongly implicate a role for natural selection (18). Furthermore, effective population sizes within lakes are large, so genetic drift should be relatively weak (19). Yet, evidence for local adaptation remains mixed. One reciprocal transplant experiment moved fish between cages in lake and stream habitats, finding higher resident fitness within one watershed, but not other watersheds (20). A second transplant experiment found that one population outperformed another in all environments (21). A third found evidence for local maladaptation: stickleback confined to cages in their native habitat (eg., lake fish in their lake, stream fish in their stream) were more heavily infected by parasites than immigrants (22). These results suggest that local maladaptation can be common (and asymmetric), potentially favoring adaptive introgression. Theory suggests that parasitism and immune evolution may be an especially potent force for such introgression (5), and stickleback have evolved substantial population differences in immune gene alleles (17), expression (23), and immune responses (24).

Here, we report a genomic time-series revealing exceptionally rapid adaptive introgression in a stickleback population from Gosling Lake, Vancouver Island. A 2005 survey of lake stickleback across Vancouver Island identified Gosling Lake as having unusually high prevalence of a tapeworm, *Schistocephalus solidus* (25). Subsequent genetic mapping revealed that Gosling Lake (GOS) stickleback had evolved infection tolerance: selection had driven fixation of a 78-bp deletion which eliminated a predicted CTCF binding site in the gene *spi1b* (24). This *spi1b*^*del*^ allele was associated with a tolerance strategy: GOS fish fail to develop fibrosis after infection, whereas the ancestral *spi1b*^*+*^ was associated with a fibrosis response that suppressed tapeworm growth. After post-glacial colonization of Gosling Lake, selection had favored *spi1b*^*del*^ over the fibrosis-prone ancestral *spi1b*^*+*^ allele, apparently reflecting a tolerance strategy (24). Although GOS were fixed for *spi1b*^*del*^ in 2009, we were surprised to find *spi1b*^*+*^ homozygotes in a 2018 sample of GOS fish caught to generate embryos. PCR reactions to detect the deletion confirmed that *spi1b*^*+*^ had increased in frequency between 2009 and 2018. We therefore tested for evolution genome-wide, by comparing sequences of 2009 versus 2022 samples from the lake (N=100 and 108 respectively). This comparison revealed large shifts in allele frequencies on all chromosomes (mean between-year F_ST_=0.175, mean allele frequency change Δp=0.227, Fig. S1), in 13 generations (or fewer). At many loci, these changes involved alleles novel to the GOS fish (absent in the 2009 sample), suggesting an effect of introgression rather than selection on standing variation.

To identify possible sources of introgression, we compared allele frequencies from the 2022 GOS fish genomes, with allele frequencies from 36 populations throughout Vancouver Island from a previous ddRAD study (18). Previously, lakes within the Campbell River watershed (including Gosling) had formed a well-supported clade, distinct from neighboring watersheds. Yet, in 2022 GOS fish were as closely related to lakes in the isolated Mohun watershed (F_ST_=0.125 between GOS and Comida Lake [COM] 9.5 km away), as they were to their immediate neighbor Boot Lake (1.3 km away, with F_ST_=0.123; Fig. S2 & S3). The recent GOS genotypes bore less affinity to other lakes in the region, suggesting introgression from the Mohun watershed (Comida and Mohun Lakes, COM and MOH). In a 2010 experiment testing for local adaptation, stickleback from COM and MOH had been transplanted into cages in Gosling Lake (23) (with provincial approval Fish Transfer Permit IT-12085). Some cages in Gosling Lake were vandalized, inadvertently releasing 37 COM and 15 MOH fish into Gosling Lake. This is a large lake (62.5 ha, 6.6 km of shoreline), so the effective population size of the residents is in the tens of thousands (19) and the census size of adults is likely in the hundreds of thousands or more (a survey of nests found >1 nest per meter of surveyed shoreline (26)). Therefore, the inadvertently introduced fish constituted less than a tenth of a percent of the resident population.

Re-sequencing additional individuals from 2007–2022 GOS collections (and five nearby populations; Table S1) confirmed that the Mohun watershed (COM and MOH) was the likely source of immigration (Fig. 1A). The timing and speed of introgression imply exceptionally strong selection, with Mohun watershed ancestry increasing dramatically between 2010 and 2013 and continued to increase thereafter (Fig. 1B). From 2005 to 2010 all sequenced fish exhibit pure GOS genotypes. In 2011 and again in 2012 we sampled one individual GOS-Mohun hybrid (Fig 1C; Fig. S4), the remaining 29 fish per year were 100% GOS (Fig. 1D). One generation later (2013) GOS-Mohun heterozygotes outnumbered native GOS homozygous genotypes on all chromosomes (Fig. 1E). By 2022 most chromosomes exhibited a majority of introgressed Mohun watershed homozygotes, though the extent of introgression varied among chromosomes (Fig. 1F). The speed of the introgression can be seen by plotting the proportion immigrant ancestry through time for each chromosome (Fig. 1G). Through 2018, there were no significant between-chromosome differences in the proportion immigrant ancestry (P>0.05), confirming that the speed of early introgression was similar genome-wide.

Normally studies of adaptive introgression reveal genomic windows of introgression: sites subject selection that drives a beneficial new allele (and closely linked sites) into the recipient population; this is a mirror image of the ‘genomic islands’ of divergence observed during population differentiation (27). However, in the earliest generations of introgression there is little time for recombination between physically linked sites or even for independent assortment of chromosomes. Consequently, for the first generations the entire genome behaves as if it were linked, a phenomenon not observable in retrospective studies of adaptive introgression’s long-term effects. Specifically, if selection of strength *s* favors an immigrant allele at one locus, even neutral loci on other chromosomes experience hitchhiking selection of strength *s/2*^*g*^ in generation *g*. The spread of Mohun watershed ancestry in the GOS population fits this model closely, with an effective selection coefficient *s*=0.29 (Fig. S5). This is exceptionally strong natural selection, favoring immigrant genotypes at one (or more) loci.

In later generations, independent assortment erodes disequilibrium between chromosomes, and the effective strength of selection on neutral loci *s/2*^*g*^ asymptotically approaches zero (28). Consistent with this theory, after ~10 generations, many chromosomes leveled off at 64% Mohun watershed ancestry. However, selection should continue to drive introgression on chromosomes carrying the beneficial immigrant variants. Conversely, any loci that originally harbored locally adapted alleles might cause their chromosomes to reverse direction back towards greater resident ancestry. We indeed see that, by 2022, the extent of introgression differed among chromosomes (Fig. 1G, Type II ANOVA, chromosome*year F_20, 5294_=4.387, P <0.001; Figs. S6&S7). Chr4 approached 80% Mohun watershed ancestry (Fig. 1H). In contrast, Chr7 reversed and became more GOS-like between 2018 and 2022 (Fig. 1I), suggesting this chromosome harbored locally adapted native alleles, that had been transiently overwhelmed by linked selection for immigrant genotypes elsewhere.

This emerging among-chromosome variation in introgression helps us identify chromosomal regions, and perhaps candidate genes, most likely to be targeted by selection for immigrant alleles. Locus-specific F_ST_ (2009 versus 2022) varied substantially across the genome ranging from 0.0 to 1.0 (Fig. 2A; median F_ST_=0.1155, mean F_ST_=0.1754, st.dev.=0.176, 90% quantile = 0.4561, 95% quantile = 0.5732, 99% quantile = 0.963, n = 1884931). Loci with especially high F_ST_ (e.g., above the 95% quantile) represent candidate targets of selection which includes many variable sites (n = 94247). The genes closest to the top 5% F_ST_ loci were enriched for molecular function ontologies related to small molecule binding (GO:0036094), catalytic activity (GO:0003824), transporter activity (GO:0005215), and trace-amine associated receptor (TAAR) activity (GO:0001594). Some of the most-divergent loci are the TAAR genes (Fig. 2A), 13 of which occur on Chr16, which shows exceptional genetic change over 10 generations (Fig. 2B). The TAAR gene family plays a role in immune function, being required for innate cells called granulocytes to chemotactically migrate towards parasites (29). TAARs function in the vertebrate gut (30) which is the site of *S. solidus* invasion in stickleback, so this gene family may play an important role in resistance during early stages of stickleback-tapeworm interactions.

Previous genetic mapping suggested that the gene *Spi1b* influences the stickleback fibrosis response to tapeworm infection (24). The *Spi1b*^*del*^ allele which was fixed in GOS fish, was genetically correlated with a suppressed fibrosis response, probably an example of evolved tolerance. Between 2005–2010, all 320 sampled GOS fish were *Spi1b*^*del*^ homozygotes (Fig. 3A, allele frequency=1.0 [0.9882, 1.0]). But, *Spi1b* was among the top 10% fastest-diverging genes after 2010 (Fig. 2A, F_ST_=0.545). Using PCR genotyping, we found the first *Spi1*^*+*^*/Spi1b*^*del*^ heterozygote in 2012 (frequency of 1.5%). In just a single year after this (one generation), the *Spi1b*^*+*^ allele frequency increased to 40% in 2013. In a decade the immigrant allele reached a frequency of 58.3% by 2022 (Fig. 3B; Table S2). In 2022, individuals with the *Spi1b*^*+*^ allele had disproportionately more COM ancestry (Fig. 3C). Although Chromosome 2 as a whole did not show exceptional introgression (Fig. 1F), local ancestry PCA (31) reveals especially high COM ancestry in the narrow chromosomal region immediately surrounding *Spi1b*, relative to the rest of Chr 2 (Fig. 3D). From this, we infer that *Spi1b* is likely to be one of the targets of selection during introgression, with the immigrant *Spi1b*^*+*^ allele partially replacing the *Spi1b*^*del*^ allele that had been inferred to provide fibrosis suppression and tolerance of *S. solidus* infection (24).

The introgression of Mohun watershed ancestry (including *Spi1b*) coincided with a steady decline in tapeworm prevalence in GOS stickleback. Tapeworm prevalence in GOS dropped from 73% (2005) to just 8% (2022) (Fig. 4A; binomial GLM, logRR=–0.18, P <0.0001), negatively associated with increased *Spi1b*^*+*^ allele frequency (Fig. 4B). Meanwhile, fibrosis increased transiently in GOS fish (Fig. 4C). Early surveys of Gosling Lake stickleback (2005, 2009, 2010, 2011) detected no fibrosis despite the high prevalence of infection. This matches previous reports that lab-raised GOS fish lacking the fibrosis response to *S. solidus* (24). However, in 2016 we were (at the time) surprised to observe severe fibrosis in 9 out of 31 sampled fish. By 2022, fibrosis was present and disproportionately found in the few fish with infections (correlation between log infection intensity and fibrosis score, r=0.449, df=143, P<0.0001) (Fig. 4D). These results confirm that GOS stickleback gained the capacity for fibrosis response, which had been absent a decade earlier (24). Plotting the trajectory of infection and fibrosis over time (Fig. 4D), we see a shift from high infection and low fibrosis (2005) to high fibrosis (2013), then reduced infection and reduced parasitism in 2014, and even less in 2022. This counter-clockwise arc in Fig. 4D matches theoretical expectations for the evolution of an inducible immune response (32): an initially susceptible population is heavily infected, but as inducible immunity evolves, infections decline. At low infection rates, the hosts retain the genetic capacity for an immune response but are no longer triggered to express the trait. As parasite abundance declines, selection for the immune trait weakens so the defensive allele stops increasing in frequency (32). This model thus can explain why selection for *Spi1b*^*+*^, though initially strong, may have weakened in the later generations.

Most studies that use outlier scans to detect targets of selection in wild populations typically do not confirm candidate genes’ function (33). However, one of our putative targets of adaptive introgression, *Spi1b*, has previously been linked to fibrosis and tapeworm resistance during studies of this same population (24). Two new experiments confirm that previous QTL association. First, we performed CRISPR/Cas9 gene editing to knock out *Spi1b* in F1 hybrid embryos from a Gosling × Roberts cross generated in 2018. All surviving F2 progeny of these F1s (n>300) were heterozygous knockouts (*Spi1b*^*+*^*/Spi1b*^*KO*^), so *Spi1b* knockouts are lethal when homozygous. When the edited fish were a year old, we used alum injections to induce fibrosis (34) in heterozygous knockout fish and wild type siblings, with saline control injections. At both 7 and 28 days post-injection (DPI) *Spi1b*^*+*^*/Spi1b*^*KO*^ knockout fish exhibited significantly elevated fibrosis compared to *Spi1b*^*+*^*/Spi1b*^*+*^ siblings (Fig. 5A&B; Alum F_₁,₇₀_=350, P <0.001; DPI F_₁,₇₀_=343, P <0.001; knockout F_₁,₇₀_=5.7, P=0.020; Alum×DPI F_₁,₇₀_=250, P <0.001). This significant effect of *Spi1b* editing confirms that this gene contributes to fibrosis, but the effect direction was opposite to our initial expectation (the 78-bp deletion having been associated with lower fibrosis). This unexpected effect direction may be due to the use of a knockout that does not recreate the 78bp deletion hypothesized to effect gene regulation. Additionally, the immune adjuvant (alum) used to induce fibrosis evokes somewhat different transcriptional pathways when compared tapeworm infection or tapeworm protein injection (35).

Pharmacological inhibition of *Spi1b*’s protein product further confirms our conclusion that *Spi1b* plays a key role in fibrosis and better fits our expected effect direction. *Spi1b* produces a protein called PU.1, a transcription factor activating fibroblasts (36). The small-molecule inhibitor db1976 blocks PU.1 activity (37), avoiding potential artifacts of gene editing. The pro-inflammatory adjuvant alum induces fibrosis in all stickleback populations regardless of genotype (34) (t=4.94, P <0.0001), and nearly all fish species (36). We co-injected a marine population of stickleback with alum (or saline controls) with varying doses of db1976 (or DMSO controls). Importantly, db1976 reduces the fibrosis response to alum in a dose-dependent manner (Fig. 5C; t=–3.83, P=0.0007). These results confirm that *Spi1b* affects sticklebacks’ peritoneal fibrosis response.

We then experimentally confirmed that fibrosis plays a causal role in tapeworm resistance. Immediately after alum injections to induce fibrosis in lab-raised Gosling Lake fish (lab raised from 2019 wild caught parents, genotype unknown) we fed individuals tapeworm-infected copepods. The pharmacologically-exaggerated fibrosis reduced both the number and mass of tapeworms compared to PBS-injected controls (Fig. 5D; Poisson GLM, Z=–2.78, P=0.005 for infection intensity; Fig. 5E; r=–0.71, P=0.035 for tapeworm mass).

Our observations document remarkably rapid genome-wide adaptive introgression caught in real time through long term genomic sampling. A small number of immigrants contributed foreign alleles into a large established population, likely in 2010. In less than a decade, the immigrant genotypes had increased to become the majority. Contrary to the common expectation in evolutionary biology that immigrants between conspecific populations will be locally maladapted in their new environment, this rapid introgression clearly shows that gene flow can supply beneficial genetic variants favored by strong natural selection. Such strong selection drives genome-wide introgression in the earliest generations, affecting all chromosomes essentially equally. This genome-wide effect is missed in retrospective studies inferring ancient admixture, which left only scattered fingerprints of Neanderthal ancestry (13). This is because over later generations, selection continued to drive introgression at some loci and chromosomes, but other chromosomes leveled off or even began to revert towards native genotypes. We infer that strong initial selection is followed by a sorting process once independent assortment and recombination sufficiently reduce linkage disequilibrium so that selection can act separately on immigrant and locally adapted loci. The targets of continued introgression included loci linked to parasite resistance. In particular, the fibroblast-activating gene *Spi1b* was previously associated with stickleback resistance to *S. solidus* tapeworms through genetic mapping (24) and transcriptomics (35). The introgression of *Spi1b*^+^ was faster than much of the rest of the genome, especially early on, and we see a corresponding increase in fibrosis and decline in *S. solidus* prevalence. We experimentally confirmed the effect of this gene through cas9 knockouts, drug inhibition, and an infection experiment. From these results we infer that part of the introgression (though by no means all) was likely driven by increased immunity to the abundant and highly virulent *S. solidus* tapeworm, whose prevalence was suppressed. Overall, our results provide a detailed case study illustrating the capacity for gene flow between allopatric populations of a given species to drive rapid adaptation, with an unusually fine-grained view of the earliest years of that introgression.

## Supplementary Material

Supplement 1

## Figures and Tables

**Figure 1:**
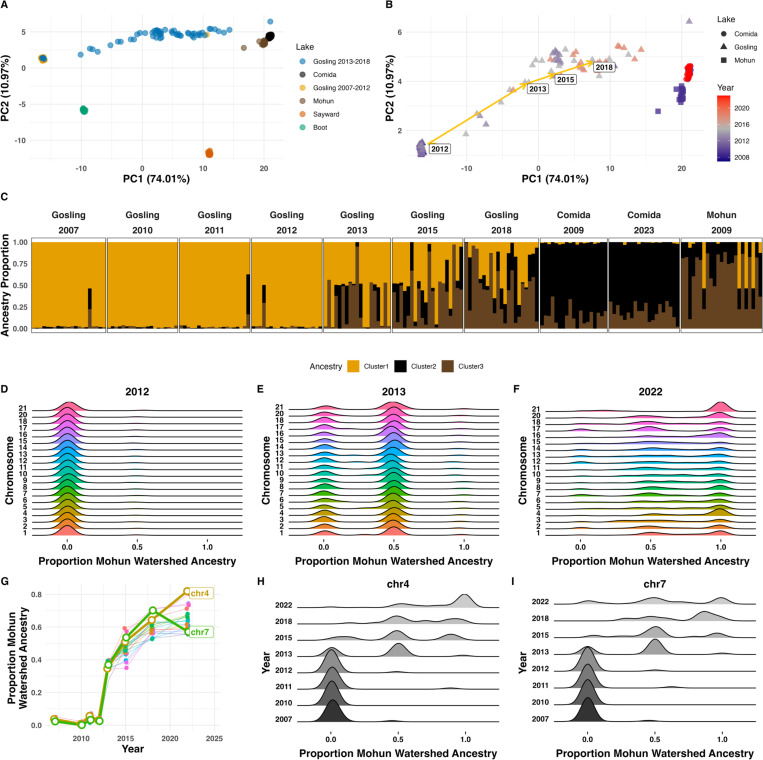
Rapid genome-wide introgression. (**A**) Genome-wide principal components analysis for Gosling (GOS) samples from 2007–2018 (lcWGS sequence data), along with Comida Lake (COM), Mohun Lake (MOH) and a sample of nearby lakes and marine outgroup (Sayward). (**B**) Time course of introgression with Gosling and Mohun watershed lakes. Labels indicate annual averages. (**C**) Admixture estimations for Gosling Lake, and the Mohun Watershed (COM, MOH) for k = 3. (**D - F**) The Mohun watershed ancestry proportion in Gosling Lake samples for each chromosome in the years 2012, 2013, 2022. (**F**) Mean proportion of Mohun watershed ancestry over time, for each chromosome. (**G-H**) Time course of Mohun watershed ancestry proportion for Chr4 and Chr7.

**Figure 2:**
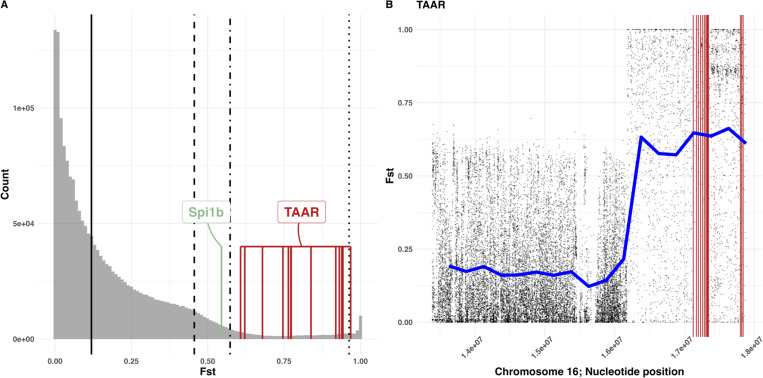
Genetic differentiation spanning the introgression event in Gosling Lake from 2009 versus 2022. (**A**) Histogram of between-year F_ST_ values. The vertical solid line is the genome-wide mean F_ST_, the dashed line indicates 90th quantile, the dash-dotted line represents the 95th quantile, and the dotted line represents the 99th quantile. The red lines indicate F_ST_ values for SNPs closest to trace-amine associated receptor (TAAR) genes (GO:0001594), the pale green line indicates F_ST_ for *Spi1b*. (**B**) Fst surrounding the TAAR loci (red) on Chr16 with smoothed F_ST_ trends in blue.

**Figure 3:**
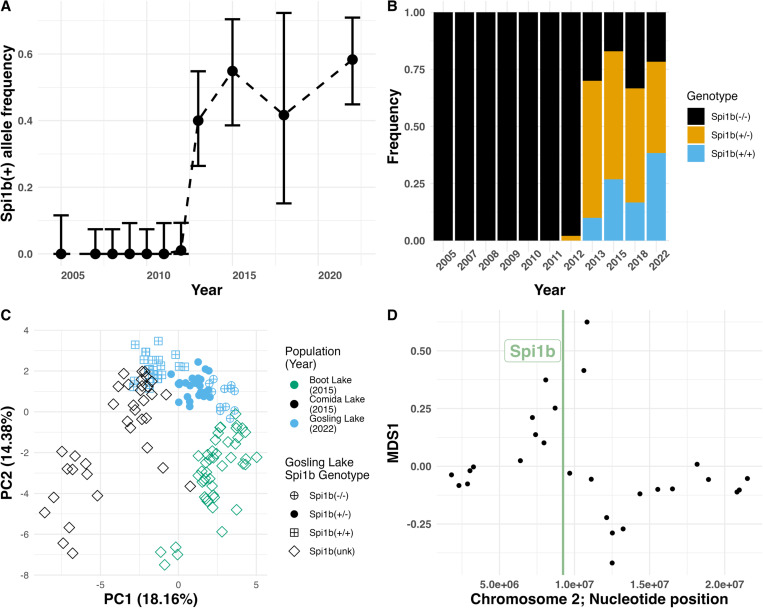
Introgression of a candidate gene, *Spi1b.* (**A**) Spi1b^+^ allele frequency change during the pulse of introgression, (**B**) genotype frequency change prior and following the introgression event, (**C**) association of *Spi1b* PCR based genotype for Gosling Lake with ancestry PCA. (**D**) the variation in structure between windows across Chr2 for Gosling Lake sampled in 2022 with Comida Lake, and Boot Lake. Each point represents a non-overlapping genomic window (mean window size 539.3kb with three loci) wherein a PCA is performed uniquely for each window then window similarity is scored using multidimensional scaling (MDS) transformation. The genomic windows adjacent to Spi1b (vertical red line) show dissimilar population structure in MDS space relative to the background structure present on Chr2.

**Figure 4.**
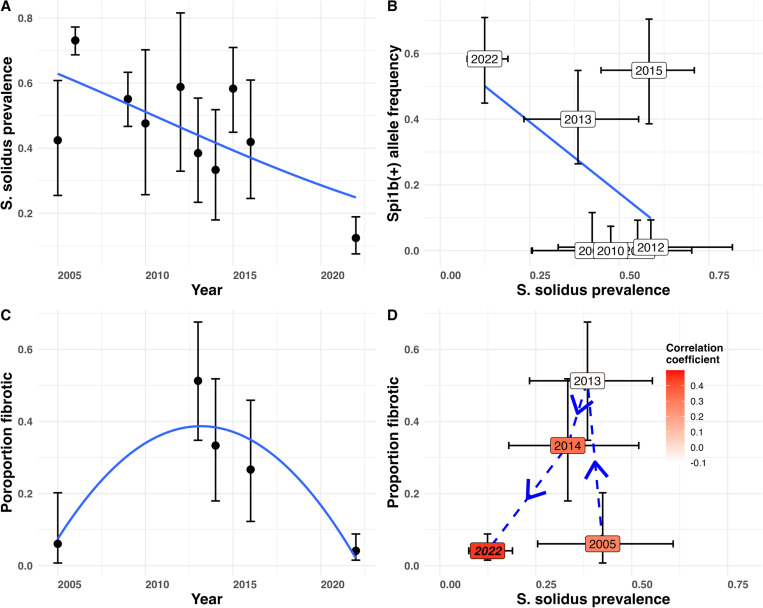
Temporal dynamics of parasite prevalence, *spi1b* with a 78-bp deletion allele frequency, and the fibrosis frequency in Gosling Lake. (**A**) From 2005 to 2022 the *S. solidus* prevalence decreased over time during the introgression. (**B**) Changes in *spi1b* reference allele frequency were associated with changes in tapeworm prevalence during the introgression period. (**C**) Peritoneal fibrosis exhibits a significant quadratic change in prevalence over time, from initially low fibrosis, to high fibrosis early in introgression, to low fibrosis when the parasite is less common. (**D**) A trajectory of the relationship between parasite prevalence and fibrosis frequency over time, informed by the immune eco-evo dynamic model presented in (32). The shading within each time point illustrates the strength of correlation between log-transformed *S. solidus* prevalence and fibrosis score. The bold italics font face in 2022 signifies a significant correlation (P <0.05).

**Figure 5:**
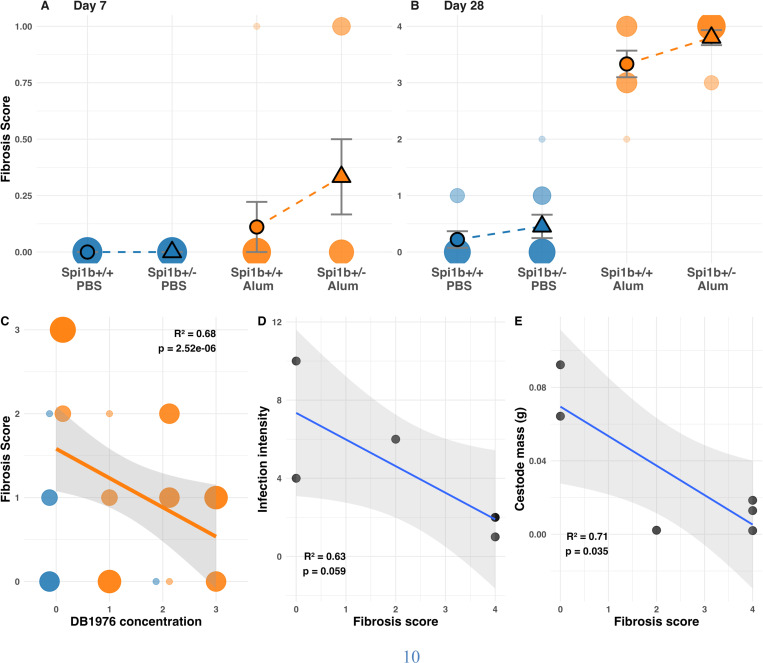
Spi1B expression regulates the fibrosis immune response in Stickleback. The association between *Spi1b* knockouts and fibrosis in response to alum or saline injections (PBS) at (**A**) 7 and (**B**) 28 days post-injection. (**C**) db1976 inhibition of *Spi1b* causes suppression of fibrosis in marine stickleback injected with alum. This inhibition of fibrosis is dose-dependent (n=8 for each concentration). Alum-induced fibrosis suppresses tapeworm infection (**D**) intensity and (**E**) mass in lab-raised GOS stickleback experimentally fed 10 tapeworms.

## Data Availability

All data required to reproduce results of this paper have been deposited in a public database (sequence data have been deposited as two NCBI BioProjects (PRJNA1303581 and PRJNA1303714); other data and R scripts are publicly available at Zenodo (DOI: 10.5281/zenodo.16969467).
