# Supplementary material for: Rapid genome-wide introgression reveals fitness advantage of immigrant genotypes
Source: bioRxiv. 2025 Aug 28:2025.08.27.672692. Preprint. [Version 1] doi: 10.1101/2025.08.27.672692 (PMC12407968; doi:10.1101/2025.08.27.672692)
Supplement: Supplement 1 [file NIHPP2025.08.27.672692v1-supplement-1.pdf]

bioRxiv preprint doi: https://doi.org/10.1101/2025.08.27.672692; this version posted August 28, 2025. The copyright holder for this preprint (which was not certified by peer review) is the author/funder, who has granted bioRxiv a license to display the preprint in perpetuity. It is made available under aCC-BY 4.0 International license.

Supplementary Materials for

Rapid genome-wide introgression reveals fitness advantage of immigrant genotypes

Ben A. Flanagan†*, Arshad Padhiar †, Foen Peng, Saif Quraishi, Andrea J. Roth- Monzón, Fahad Gilani, Lauren Simonse, Meghan Maciejewski, Noah Reid, Milan Malinsky, Amanda K Hund, Daniel I. Bolnick

* Corresponding author. Email: benjamin.flanagan@uconn.edu

† Equal contributions to authorship

The PDF file includes:

Materials and Methods
Figs. S1 to S7
Tables S1 to S4
References

bioRxiv preprint doi: https://doi.org/10.1101/2025.08.27.672692; this version posted August 28, 2025. The copyright holder for this preprint (which was not certified by peer review) is the author/funder, who has granted bioRxiv a license to display the preprint in perpetuity. It is made available under aCC-BY 4.0 International license.

Materials and Methods

Field collections

Wild caught threespine stickleback (*Gasterosteus aculeatus*) on Vancouver Island were collected using unbaited minnow traps set in the littoral zone during late May to early June across a 17-year period between 2005 to 2022 (years and sample sizes in Table S1). All collections were conducted under approval from the University of Texas at Austin IACUC (protocol AUP- 03120501, AUP-05111701, AUP-07-032201 AUP-2010-00024, AUP-2015-00200), and subsequently the University of Connecticut IACUC (protocol A-18-008, A-21-025) and Scientific Fish Collection Permits from the Ministry of the Environment of British Columbia (NA05-19046, NA06-21423, NA07-32612, NA08-43012, NA09-34960, NA10-61026, NA11-70031, NA12-77018, NA13-85103, NA14-93580, NA15-16384, NA15-217759, NA18-287894, NA22-679623). Further details of the survey, including collection location coordinates are provided in (*18*, *24*, *25*, *38*). Upon capture, fish were euthanized in the field in MS-222. Fin clips were retained in ethanol, enabling subsequent genotyping work (see below). Specimens were either dissected when freshly caught (e.g., in 2022), or more typically were preserved in 10% neutral buffered formalin for subsequent dissection.

Sample collection for DNA extraction and genotyping

Upon capture, fish were euthanized in the field, and a small portion of the caudal fin was clipped, placed in 95% ethanol, and stored at –20°C until DNA extraction. Fin clips used for whole genome sequencing were collected from fish sampled across the following lake-year combinations: Boot 2023, Comida 2009 and 2023, Gosling 2007, 2010, 2011, 2012, 2013, 2015, 2018, and 2022, Higgens 2009, Mohun 2009, Roberts 2013, and Sayward 2009. DNA was extracted using the Qiagen DNeasy Blood & Tissue Kit (catalog #69506), following the manufacturer's instructions. For certain lab-raised individuals used in genotyping assays (e.g., validation of *spi1b* knockouts), DNA was extracted from freshly clipped fin tissue using the same protocol.

High Coverage Sequencing of 2022 Gosling Lake Samples

We performed high-coverage whole genome sequencing (WGS) on 108 individual threespine stickleback collected from Gosling Lake in 2022. DNA was extracted from ethanol-preserved fin clips as described above, and libraries were prepared using the Illumina Nextera DNA Flex Library Prep Kit. Samples were pooled and sequenced in a single lane of an Illumina NovaSeq S4 v1.5 flow cell (300 cycles) at the Center for Genome Innovation (CGI), University of Connecticut, generating 150 bp paired-end reads. The sequencing design targeted an average depth of approximately 12–15× coverage per individual.

Reads were aligned to the *G. aculeatus* v.5 reference genome (*39*) using bwa -mem v.0.7.17 (Li, 2013). To tag PCR and optical duplicate reads we used the MarkDuplicates tool from the Picard package v.2.27.4. Variant calling was performed with GATK v.4.2.3 (DePristo et al., 2011), using HaplotypeCaller in GVCFmode for each individual separately, followed by joint genotyping using GenotypeGVCFs with the --include-non-variant-sites option. For variant filtering we generated a 'callability mask', identifying the genomic regions where we were unable to call variants with confidence. The callability mask consisted of four elements: (i) sites with unusually high or unusually low overall coverage, based on examining coverage histograms; (ii) sites where more than 10% of individuals had missing genotypes; (iii) sites identified by GATK as low quality (i.e., with the LowQual tag) and (iv) sites with 'poor mappability'. Mappability was assessed by breaking down the genome into overlapping k-mers of 150bp (matching the read length) and mapping these k-mers back to the genome. Using the SNPable tool (http://lh3lh3.users.sourceforge.net/snpable.shtml), we then masked all sites where fewer than 90% of k-mers mapped back to their original location perfectly and uniquely. On autosomes, the callability mask comprised 94.8Mb, which is about 22.8% of sequence. After applying the callability mask, we added several hard filters based on GATK best practices: specifically focusing on mapping quality (MQ < 40), mapping strand bias (FS > 40), variant quality normalized by depth (QD < 2) and excess heterozygosity when compared with Hardy–Weinberg equilibrium (ExcessHet > 40).

To estimate the admixture proportions for the 108 Gosling Lake fish from 2022, we used PLINK v1.90b4.4 (*40*) with the resultant chromosome-specific VCF file as input and removed variable sites which had greater than 5%

bioRxiv preprint doi: https://doi.org/10.1101/2025.08.27.672692; this version posted August 28, 2025. The copyright holder for this preprint (which was not certified by peer review) is the author/funder, who has granted bioRxiv a license to display the preprint in perpetuity. It is made available under aCC-BY 4.0 International license.

missing genotype call rate and only retained biallelic SNPs. Then genotype matrices were generated using PLINK v1.90b4.4 (*40*), and these genotypes were used to estimate allele frequencies and these data were combined with previously estimated allele frequencies from Gosling Lake sampled in 2009 (*24*) to calculate locus-specific Fst. For the top 5% most highly differentiated loci we ran a gene ontology enrichment analysis using the online platform g:Profiler (*41*) by, first, identifying the genes nearest the highly differentiated loci using bedtools (*42*). Then, because *Danio rerio* has more functional information than *G. aculeatus*, we identified orthologous genes in zebrafish and performed ontology enrichment analysis using the zebrafish orthologs. Then 2022 Gosling Lake genotypes were combined with previous ddRADSeq genotyping effort from Boot Lake in 2013 which is adjacent to Gosling Lake, and Comida Lake from 2013 (*43*). Then we performed a local PCA (*31*) to determine how population structure changes along the genome.

<u>Low Coverage Sequencing of Time Series Samples</u>

We performed low-coverage whole genome sequencing (lcWGS; (*44*)) on 20 individuals per population from 15 lake-year combinations across Vancouver Island, including a time series from Gosling Lake spanning 2007 to 2018. Additional samples were collected from Boot, Comida, Higgens, Mohun, and Roberts Lakes, and Sayward Estuary; coordinates are provided in (*38*). Genomic DNA was extracted, (as mentioned above) and libraries were prepared using the Illumina Nextera DNA Flex Library Prep Kit, followed by sequencing on an Illumina NovaSeq S4 platform (paired-end 150 bp reads) at the Center for Genome Innovation (CGI), University of Connecticut. Sequencing targeted an average depth of ~2X per individual.

Parsed paired-end reads assigned to individuals were mapped to the *G. aculeatus* reference genome (v.5 assembly; (*39*)) using BWA-MEM (*45*). Duplicate reads in the resultant alignments were identified and discarded using samblaster (*46*), and aligned reads were sorted, compressed, and indexed using samtools (*47*). Genotype likelihoods were computed in a probabilistic framework using ANGSD (*48*), implementing the GATK genotype likelihood model (-GL 2). Variable sites were identified via a likelihood ratio test, retaining sites with a significance threshold of $p < 1e{-6}$. We generated a genome-wide Beagle-format file, which was then split by chromosome for downstream analysis. To estimate ancestry and population structure, we applied PCAngsd (v1.35; (*49*)). Admixture proportions were calculated for each chromosome individually (K = 2), and a genome-wide covariance matrix was also computed from the same Beagle file, retaining the top four eigenvectors for principal components analysis. PCAngsd estimated covariance matrix was used to perform a principal components analysis in R using the prcomp() function with a zero centered variable shift and scaled to unit variance. Then, to describe the genomic changes in Gosling Lake over time, we subset the prcomp() output to include Gosling Lake and Mohun watershed. Then for the Gosling Lake samples after the Mohun watershed fish introduction (> 2011), we calculated the mean PC1 and mean PC2 for each year. The Gosling Lake and Mohun watershed subset were then plotted as well as the year mean PC values with arrows connecting the estimated means. Then to more closely identify the introduction source, Admixture proportions (k = 3) were estimated for Gosling Lake fish from 2007 to 2018 and fish from the Mohun watershed including Comida Lake and Mohun Lake. We also estimated the covariance matrix for Mohun watershed popuoatio0ns and Gosling Lake fish from 2018, after the invasion. The subsequent covariance matrix was used to perfume a principal components analysis in R as described above and the first 40 PCs were used to perform a linear discriminate analysis in R using MASS::lda() with Mohun watershed populations (Comida Lake or Mohun Lake) as grouping variables. Then we predicted assignment to Comida or Mohun Lake for the 2018 Gosling Lake samples for all chromosomes individually. For the 2022 Gosling Lake samples the output .bed files were used as input for ADMIXTURE Version 1.3.0 (D.H. Alexander, J. Novembre, and K. Lange. Fast model-based estimation of ancestry in unrelated individuals. *Genome Research*, 19:1655–1664, 2009.) with K = 2. The PCAngsd and ADMIXTURE estimated admixture proportions for each chromosome were plotted using ggplot2::geom_density_ridges() for 2012, 2013 and 2022. Then for chr4 and chr7, the admixture proportions were plotted using ggplot2::geom_density_ridges() across years. The direction of Mohun watershed ancestry was inferred by including Comida Lake samples in the admixture proportion estimates.

We then plotted the trend in mean Mohun watershed ancestry for each chromosome across years. From this we also calculated a genome-wide average Mohun watershed ancestry for each year. This genome-wide trend was used to estimate the effective strength of selection acting on an average site in the genome. We fit a curve in which the between-year change in ancestry proportion should be equal to $s/2^g$. Sampling a wide range of possible selection coefficients s, we calculated the sum of squares as a goodness of fit between the model prediction and the observed change in genome-wide mean ancestry. The best estimate of s is the value that minimized these sums of squares.

bioRxiv preprint doi: https://doi.org/10.1101/2025.08.27.672692; this version posted August 28, 2025. The copyright holder for this preprint (which was not certified by peer review) is the author/funder, who has granted bioRxiv a license to display the preprint in perpetuity. It is made available under aCC-BY 4.0 International license.

Within each sample year, we used an ANOVA to test whether the observed ancestry proportion differed among chromosomes. This allowed us to evaluate whether some chromosomes were, on average, introgressing faster or slower than others. Likewise, we used t-tests to evaluate whether a given chromosome increased (or, decreased) its ancestry proportion between years.

Changes in *Spi1b*^{del} frequency

Weber et al. (***24***) described a deletion in the gene *spi1b* that was associated with loss of fibrosis and increased tolerance of *S. solidus* infection in Gosling Lake stickleback. To assay changes in this focal gene over time, targeted genotyping to detect the *Spi1b*^{del} deletion allele was performed using PCR with the following primers: forward 5′–tcactgagaaagcgccagtt–3′ and reverse 5′–tgtttcatggatgaatgaag–3′. The expected product size was 825 bp for wild-type alleles (*Spi1b+*) and 747 bp for alternate allele that had 78 bp intronic deletion (*Spi1b*^{del}). PCR products were run on an agarose gel, stained with Cybersafe, and photographed. Comparison with a standard ladder allowed us to score individuals as wild type homozygote, deletion homozygote, or heterozygote. We used this PCR protocol to score *spi1b* genotype on individuals from 2007 through 2022 (sample sizes listed in Table S1). We used a binomial GLM to test for changes in allele frequency between years, and over the duration of the study (time as a numeric variable). The 2022 Gosling Lake called genotypes were combined with Comida Lake and Boot Lake genotyped using ddRADseq by Stuart et al (*18*). We then performed a principal components analysis as previously described with the fill of the points was based on *Spi1b* PCR genotypes.

Functional validation of *Spi1b* role in fibrosis response: Spi1b knockout

CRISPR/Cas9 Knockout of Spi1b To disrupt *Spi1b*, we designed two sgRNAs targeting exon 2 using CHOPCHOP (*50*, *51*) based on the *G. aculeatus* BROADS1 genome assembly. The sgRNAs (5′–cgctcaccctcacgttcacc–3′ and 5′–acaggacgccatacggtacg–3′) were ordered as crRNAs from Integrated DNA Technologies (IDT). Each sgRNA was tested separately in independent microinjection experiments, and both produced comparable knockout efficiency.

RNP complexes were prepared by combining crRNA, tracrRNA, and Cas9 protein (all from IDT). Duplex buffer was used to make 100 µM stock solutions of individual crRNAs and tracrRNA. To generate crRNA:tracrRNA duplexes (50 µM), equal volumes of crRNA and tracrRNA were mixed and annealed in a thermal cycler: 95°C for 5 minutes, cooled to 25°C at 0.1°C/s, held at 25°C for 5 minutes, and then held at 4°C. The resulting 50 µM duplex was diluted to 25 µM for use in injection mixes and stored at –20°C. Injection cocktails (4 µL) were prepared fresh on the day of injection and included 0.4 µL 25 µM crRNA:tracrRNA duplex, 1.2 µL 50 µM Cas9 protein, 1.2 µL nuclease-free water, and 0.4 µL of 0.5% phenol red. Control cocktails (4 µL) contained 1.2 µL Cas9 protein, 2.4 µL water, and 0.4 µL phenol red, maintaining Cas9 concentration identical to that of treatment groups. Each mix was incubated at 37°C for 5 minutes before microinjection and kept at room temperature. Approximately 2 nL of the injection mix was injected into the yolk or cytoplasm of one-cell stage embryos using borosilicate capillaries.

Microinjections were performed following in vitro fertilization. Stickleback eggs were obtained from mature females and fertilized using sperm prepared by macerating male testes in Hank's solution. Up to 100 eggs were combined with 50 µL of sperm solution to ensure fertilization. Embryos were kept covered to prevent drying during early development. Injection materials were prepared while embryos developed to the one-cell stage, typically within 20–25 minutes. Following injection, embryos were transferred to Petri dishes containing stickleback water (prepared from artificial seawater mix, 10% sodium bicarbonate, and deionized water). All procedures were performed at room temperature. The injected embryos were F1 hybrids generated from a cross between Gosling and Roberts Lake fish collected in 2018. These injected F1s, which were expected to be mosaic for CRISPR-induced mutations, were raised to maturity and crossed to produce F2 offspring. We screened F2 individuals for *Spi1b* knockouts using gel band shift PCR followed by Sanger sequencing. Despite extensive screening, we recovered only monoallelic knockouts; no biallelic knockouts were identified. This may reflect an essential role for *Spi1b* during development, as complete knockout could be lethal. This interpretation is consistent with prior reports of lethality in *Spi1* knockout mice (*52*). Additionally, we observed elevated mortality in mosaic crosses, further suggesting potential developmental constraints on complete gene loss. Genotyping was performed using the following primers: forward 5′–ggtgatttctgtcctgttttgga–3′ and reverse 5′–gtgcacgaaggtcatgaagc–3′.

Testing Fibrosis Response in Spi1b KO Fish. To test whether *Spi1b* influences the fibrosis response, we injected F2

bioRxiv preprint doi: https://doi.org/10.1101/2025.08.27.672692; this version posted August 28, 2025. The copyright holder for this preprint (which was not certified by peer review) is the author/funder, who has granted bioRxiv a license to display the preprint in perpetuity. It is made available under aCC-BY 4.0 International license.

monoallelic knockouts and non-knockout siblings with either saline (PBS) or alum. Intraperitoneal injections consisted of 20 µL total volume: either 20 µL PBS or 10 µL of 2% Alumax Phosphate (OZ Biosciences) mixed with 10 µL PBS. Alum is a widely used immune adjuvant that induces leukocyte recruitment and peritoneal fibrosis ((*53*); N. Steinel, pers. comm). For each genotype, 9 fish were injected with PBS and 9 with alum. Fish were sampled at 7 and 28 days post-injection. Fibrosis scores from days 7 and 28 were analyzed using linear models with fixed effects for treatment (alum vs. PBS), genotype (monoallelic Spi1b knockout vs. wild type) and dissection timepoint. Interaction terms were included to assess whether treatment and genotype effects varied across timepoints, and whether the genotypes exhibited different responses to treatment. AIC model selection was used to simplify the model to a subset of empirically justified terms.

Scoring fibrosis: Fibrosis was evaluated during dissection by assessing adhesion formation within the peritoneal cavity, following the protocol first established by Hund et al (*34*). In healthy stickleback, visceral organs move freely and are assigned a fibrosis score of 0. Fibrosis is characterized by adhesions between organs or between organs and the peritoneal wall. Severity was scored on an ordinal scale from 0 to 4, where 1 indicated mild adhesions limiting organ movement, 2 indicated adhesions between organs, 3 indicated adhesions between organs and the peritoneal wall, and 4 indicated severe adhesions making dissection of the cavity difficult. Previous assays confirmed that these ordinal scores are highly repeatable among independent observers ($r > 0.9$; (*54*)).

<u>Functional validation of *Spi1b* role in fibrosis response: pharmacological Spi1b inhibition</u>

Stickleback fish from the Kenai River Flats (KRF) population (Alaska) initiate irreversible fibrosis within days of alum injection, and this fibrosis persists for up to one year. Lab-raised KRF adult fish were divided into six treatment groups, with seven individuals per group. Three groups received intraperitoneal injections of the *Spi1b* inhibitor DB1976 (*37*) at doses of 1 nM, 2 nM, or 4 nM, each co-injected with alum on day 0. A fourth group received alum co-injected with DMSO to serve as a vehicle control for the inhibitor treatments. A fifth group received two 10 µL injections of DMSO alone to control for injection number. All fish were assessed for fibrosis development on day 11 post-injection. Fibrosis was scored as described above. Fibrosis severity was analyzed using a linear model with DB1976 dose as a predictor among alum treated group. We also fit models including both alum treatment and DB1976 dose to assess their individual and combined effects on fibrosis.

<u>Testing whether alum-induced fibrosis contributes to *S. solidus* resistance.</u>

Lab raised stickleback from Gosling Lake (bred in 2018) were experimentally exposed to *S. solidus* as described in detail in (*24*, *55*). Briefly, live tapeworms were obtained from naturally infected Gosling Lake stickleback collected in the field and shipped to the University of Connecticut. Tapeworms were dissected from euthanized hosts, and size-matched pairs were placed in nylon biopsy bags suspended in breeding media in a dark, warm, shaking water bath. Eggs that passed through the mesh were collected from the bottom of the breeding jars and stored at 4°C. Eggs were hatched at 17°C under a 12-hour light cycle and resulting coracidia were used to infect *Macrocyclops albidus* copepods. Two weeks after exposure, individual copepods were screened under a dissecting microscope, and only those visibly infected were used for fish exposures.

A total of 118 Gosling Lake stickleback were distributed into two tanks and assigned to one of two treatment groups: fibrosis-induced (via alum injection) or control (PBS injection). Injections were administered one week prior to parasite exposure. Fish in the fibrosis group received an intraperitoneal injection of 10 µL 2% Alumax Phosphate (OZ Biosciences) mixed with 10 µL PBS. Control fish received 20 µL PBS. Fish were food-deprived for 24 hours before infection. On the day of exposure, water flow was paused and each fish was offered 10 infected copepods. Fish were left to feed for 6–8 hours in static water, after which circulation was resumed. Copepod consumption was not monitored individually, but tanks were filtered after the feeding period to confirm uptake. All fish were dissected two months post-infection. During dissection, fibrosis was scored as described above. Parasite load (infection intensity) and tapeworm mass (total and individual) were recorded. In cases where tapeworm mass fell below the limit of scale detection (<0.0001 g), individuals were assigned a mass of 0.00005g. Linear models were used to test whether total tapeworm mass depended on fibrosis severity. A Poisson generalized linear model (GLM) was used to test whether infection intensity depended on fibrosis score.

bioRxiv preprint doi: https://doi.org/10.1101/2025.08.27.672692; this version posted August 28, 2025. The copyright holder for this preprint (which was not certified by peer review) is the author/funder, who has granted bioRxiv a license to display the preprint in perpetuity. It is made available under aCC-BY 4.0 International license.

Changing infection prevalence and fibrosis through time

Freshy caught or archived formalin-preserved stickleback were dissected to count *S. solidus* parasites. Prior to dissection we measured individuals' standard length and body mass. During dissection we also recorded sex based on gonad anatomy. We include previously published data on *S. solidus* prevalence (*24*), supplemented with additional years of samples from archived stickleback specimens, and more recent fresh caught samples. To test for a directional temporal trend in infection prevalence, we fit a binomial generalized linear model (GLM) with year as a continuous predictor of the number of infected individuals out of the observed sample per year. We also tested for a correlation between tapeworm prevalence and *Spi1b*^{del} allele frequency.

We dissected and scored fibrosis from archived formalin-preserved samples of stickleback originally collected 2005 and 2014, and fresh-caught samples collected in 2016 and 2022. We used the ordinal scale to score fibrosis as described above. Infection intensity was also noted for each individual. We used linear and quadratic regression to test whether mean fibrosis score exhibited a directional or curvilinear trend through time. We then tested whether mean fibrosis was related to *S. solidus* prevalence, treating each year as an observation and considering both linear and quadratic relationships. *A priori* we considered a quadratic relationship likely based on a recent theoretical model (*56*). Within each year, we used a correlation test to evaluate whether fibrosis severity was related to infection intensity. Our expectation was that before introgression, fibrosis should be mostly absent and unrelated to infection (consistent with prior work by Weber et al (*24*). However, once the immigrant *Spi1b*$^{+}$ genotype invaded we expected to find a positive relationship between infection and fibrosis, consistent with laboratory evidence that infection induces fibrosis.

bioRxiv preprint doi: https://doi.org/10.1101/2025.08.27.672692; this version posted August 28, 2025. The copyright holder for this preprint (which was not certified by peer review) is the author/funder, who has granted bioRxiv a license to display the preprint in perpetuity. It is made available under aCC-BY 4.0 International license.

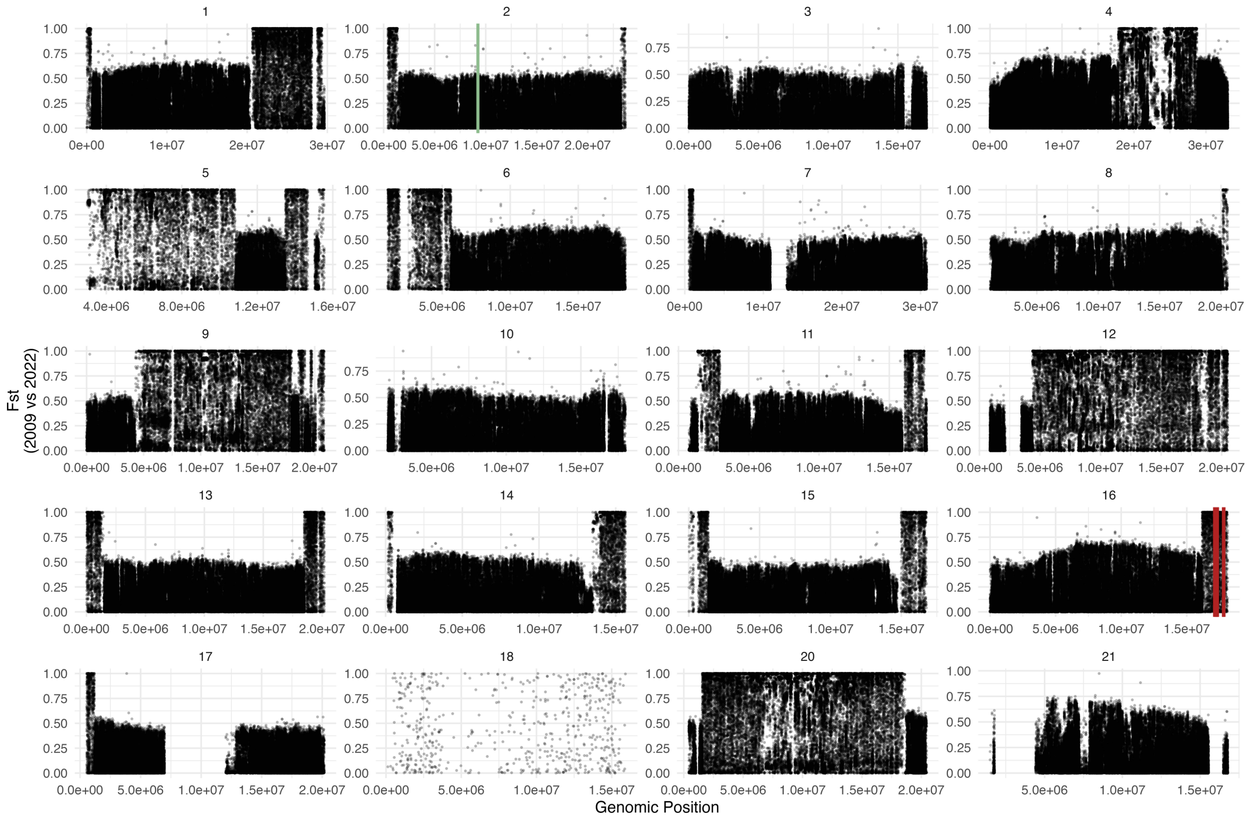

Figure S1: Genome-wide allele frequency change between 2009 and 2022 Gosling Lake stickleback. Genetic divergence in allele frequencies (F_{ST}) between Gosling Lake stickleback sampled in 2009 (N = 100 individuals genotyped by PoolSeq to obtain allele frequencies at an average of 2x depth), versus Gosling Lake stickleback sampled in 2022 (N = 108 whole genome sequences, at an average of 10x depth). Each panel is a chromosome, omitting the Y and X chromosomes, with Spi1b genomic position indicated in green on Chromosome 2 and Trace-amine associated receptor loci in red on Chromosome 16.

bioRxiv preprint doi: https://doi.org/10.1101/2025.08.27.672692; this version posted August 28, 2025. The copyright holder for this preprint (which was not certified by peer review) is the author/funder, who has granted bioRxiv a license to display the preprint in perpetuity. It is made available under aCC-BY 4.0 International license.

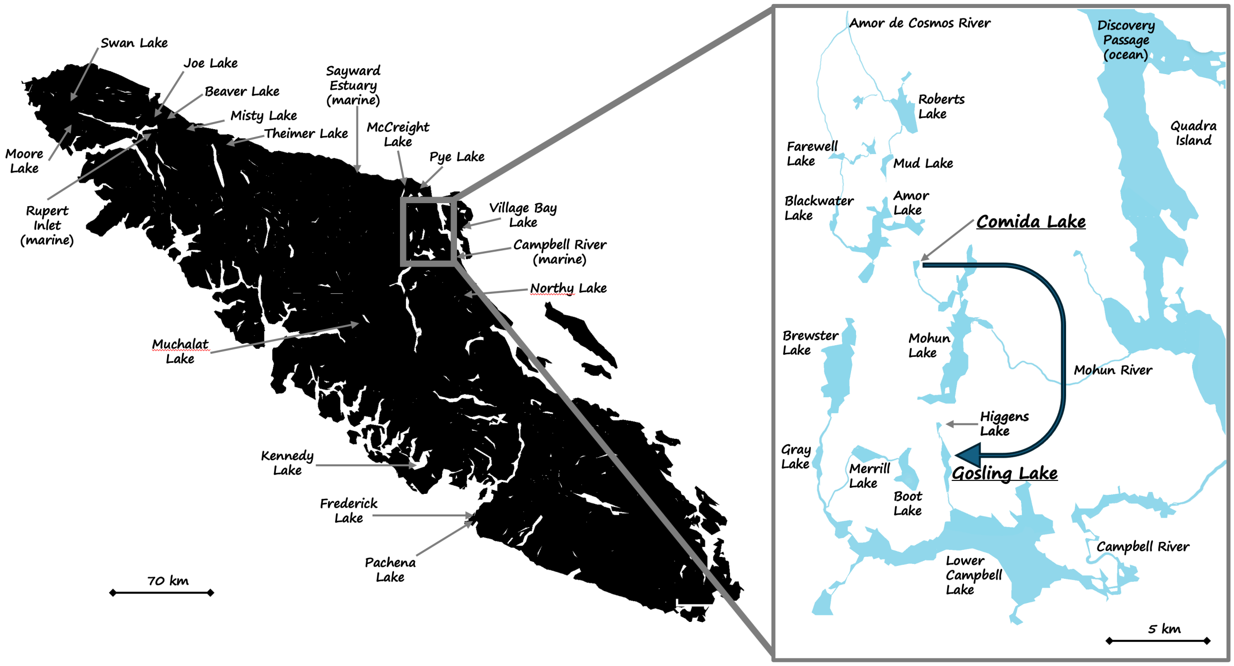

Figure S2: A map of Vancouver Island showing the populations examined here with a detailed inset. For the larger map of the island, we provide names and arrows pointing to the populations genotyped using ddRADseq by Stuart et al (*18*), which we use to evaluate possible sources of introgression. A complete phylogeny of these populations can be seen in (*18*). In the inset we show a subset of the lakes in the region, which are divided into three major watersheds: Amor de Cosmos River draining north, Mohun River, and Campbell River. Gosling Lake, the focal population in this study, is indicated with larger font, and is in the Campbell River drainage. Comida Lake, the source of migrants (also larger font), is in the Mohun River drainage. Roberts Lake (top) is in the Amor de Cosmos drainage and was used in previous crosses with Gosling Lake fish that identified *spi1b* as the likely genetic basis of variation in fibrosis based immunity to *S. solidus* infections.

bioRxiv preprint doi: https://doi.org/10.1101/2025.08.27.672692; this version posted August 28, 2025. The copyright holder for this preprint (which was not certified by peer review) is the author/funder, who has granted bioRxiv a license to display the preprint in perpetuity. It is made available under aCC-BY 4.0 International license.

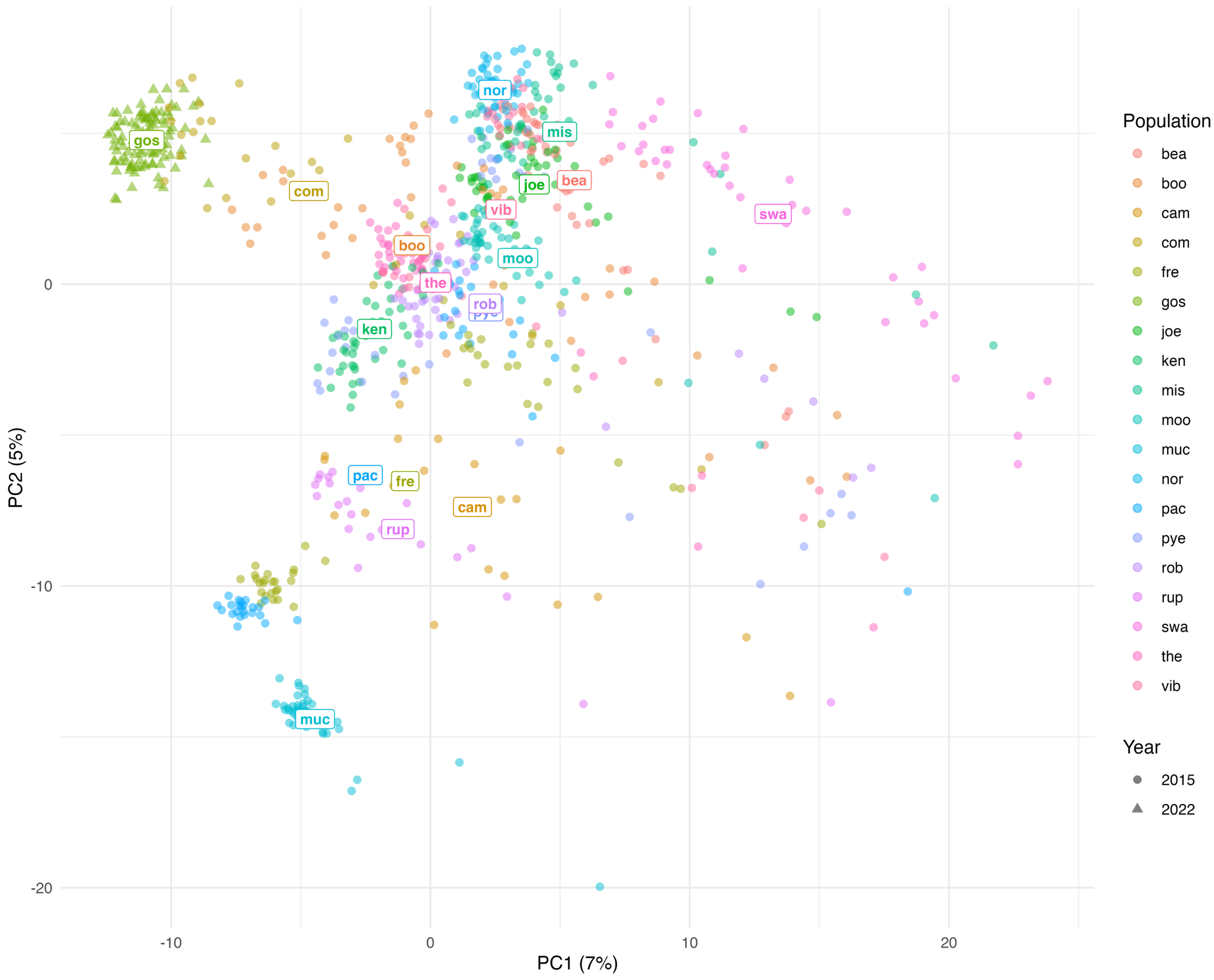

Figure S3: Principal component analysis of population differentiation using 2022 genomic SNPs and 2015 ddRAD data. Population differentiation PCA using 2022 genomic sequence SNPs and ddRAD data from 2015 (from the study by Stuart et al, (*43*)). All populations shown here are represented in the map in Fig. S2: bea = Beaver Lake, boo = Boot Lake, cam = Campbell River Estuary marine fish, com = Comida Lake, fre = Frederick Lake, gos = Gosling Lake, joe = Joe Lake, ken = Kennedy Lake, mis = Misty Lake, moo = Moore Lake, muc = Muchalat Lake, nor = Northy Lake, pac = Pachena Lake, pye = Pye Lake, rob = Roberts Lake, rup = Rupert Inlet marine fish, swa = Swan Lake, the = Theimer Lake, vib = Village Bay Lake. GPS coordinates for all populations are listed in data supplements in (*18*).

bioRxiv preprint doi: https://doi.org/10.1101/2025.08.27.672692; this version posted August 28, 2025. The copyright holder for this preprint (which was not certified by peer review) is the author/funder, who has granted bioRxiv a license to display the preprint in perpetuity. It is made available under aCC-BY 4.0 International license.

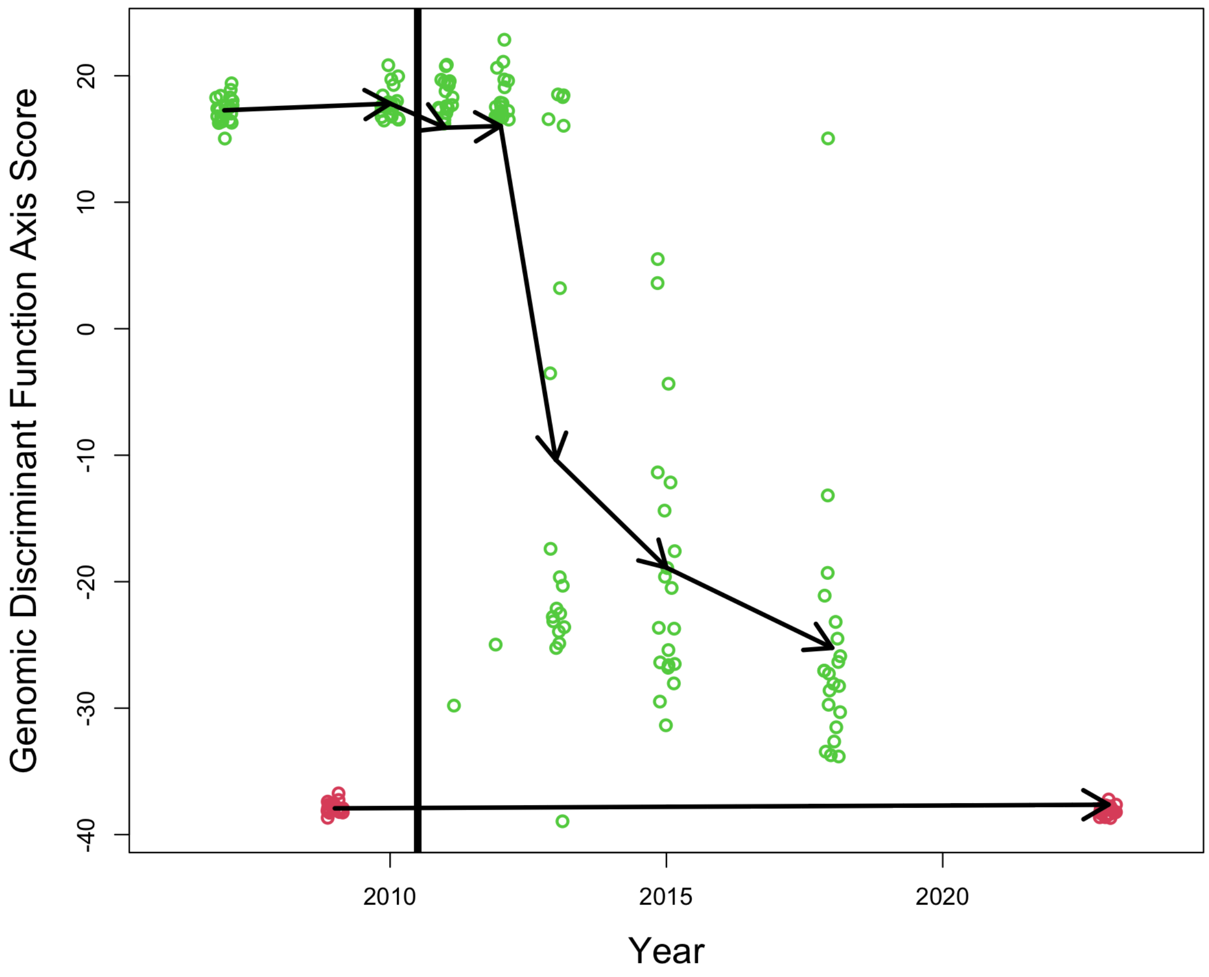

Figure S4: Discriminant function analysis reveals Comida Lake introgression into Gosling Lake over time. Alternative visualization of introgression. The red points are Comida Lake individuals, and green points are Gosling Lake individuals. We trained a discriminant function analysis based on pre-2011 samples to distinguish the two lakes, then applied this function to post-2011 samples to predict their ancestry contributions. This analysis revealed one individual fish sampled in 2011 and one in 2012 with Comida Lake ancestry contributions. In 2013, there is a mix of pure Gosling and hybrid individuals. But by 2015 no pure Gosling genotypes were sampled (possibly one sampled in 2018), revealing the systemic spread of the foreign Comida Lake genotypes.

bioRxiv preprint doi: https://doi.org/10.1101/2025.08.27.672692; this version posted August 28, 2025. The copyright holder for this preprint (which was not certified by peer review) is the author/funder, who has granted bioRxiv a license to display the preprint in perpetuity. It is made available under aCC-BY 4.0 International license.

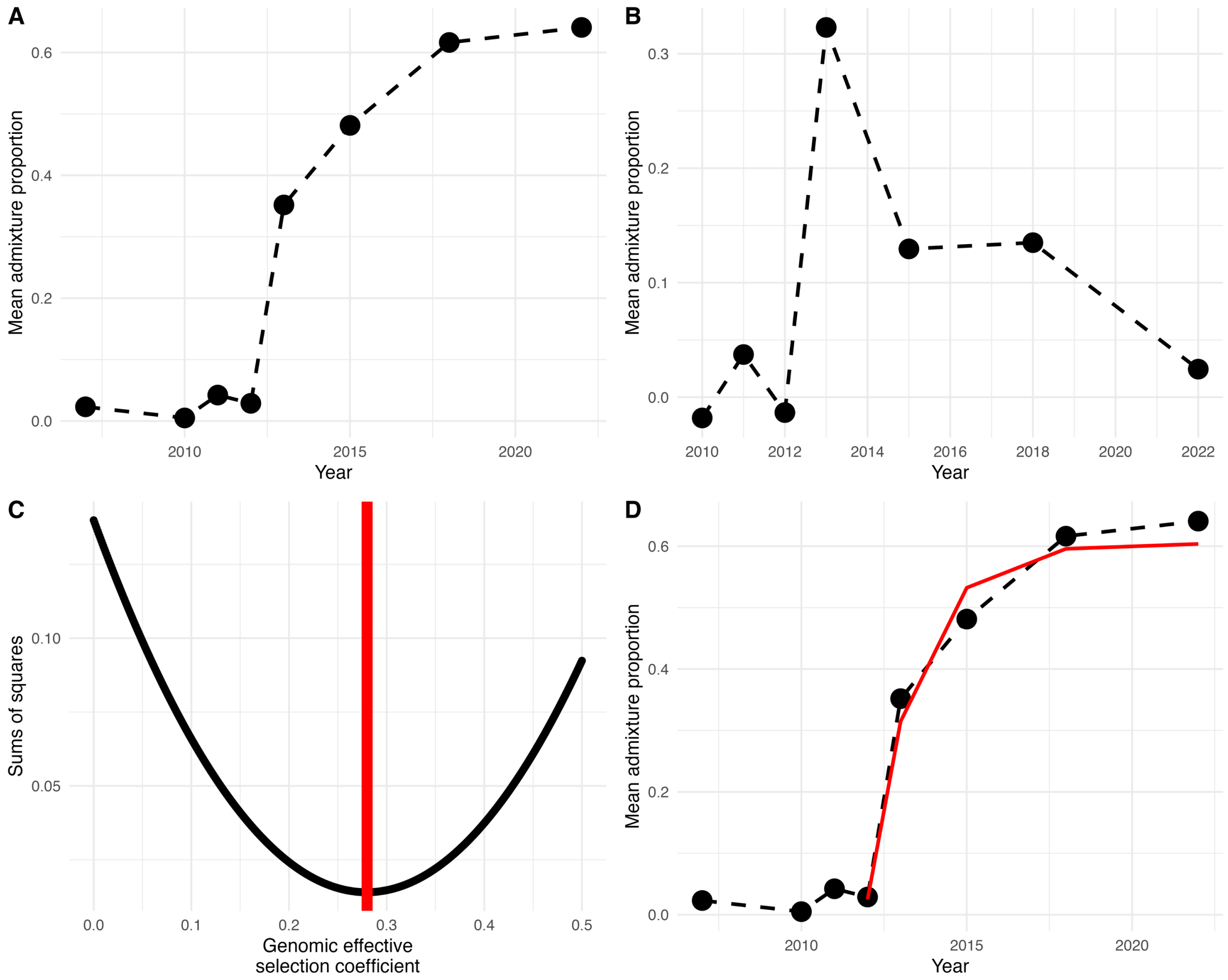

Figure S5: Estimating genome-wide effective selection coefficients. A) Observed data on the estimated admixture proportion (% Mohun watershed ancestry), averaged across the genome and across individuals. B) Observed change in admixture proportion between time points. C) We fit a model with the change in admixture proportion between successive years equal to $s/2^g$. That is, in the first generation the entire genome experiences selection coefficient *s*. In the next generation, s/2, then s/4, then s/8. Where we missed a year of sampling, the observed change in admixture proportion would be the sum of the expected values. We then calculated the sums of squares as a measure of goodness of fit between our model, and the observed values in (B), for a variety of values of *s*. We plot a vertical red like at the estimate of *s* that minimizes the sums of squares (maximum fit to the data). (D) We then replot the data from panel (A) with the modelled allele frequency change for a neutral locus experiencing hitchhiking overlain in red.

bioRxiv preprint doi: https://doi.org/10.1101/2025.08.27.672692; this version posted August 28, 2025. The copyright holder for this preprint (which was not certified by peer review) is the author/funder, who has granted bioRxiv a license to display the preprint in perpetuity. It is made available under aCC-BY 4.0 International license.

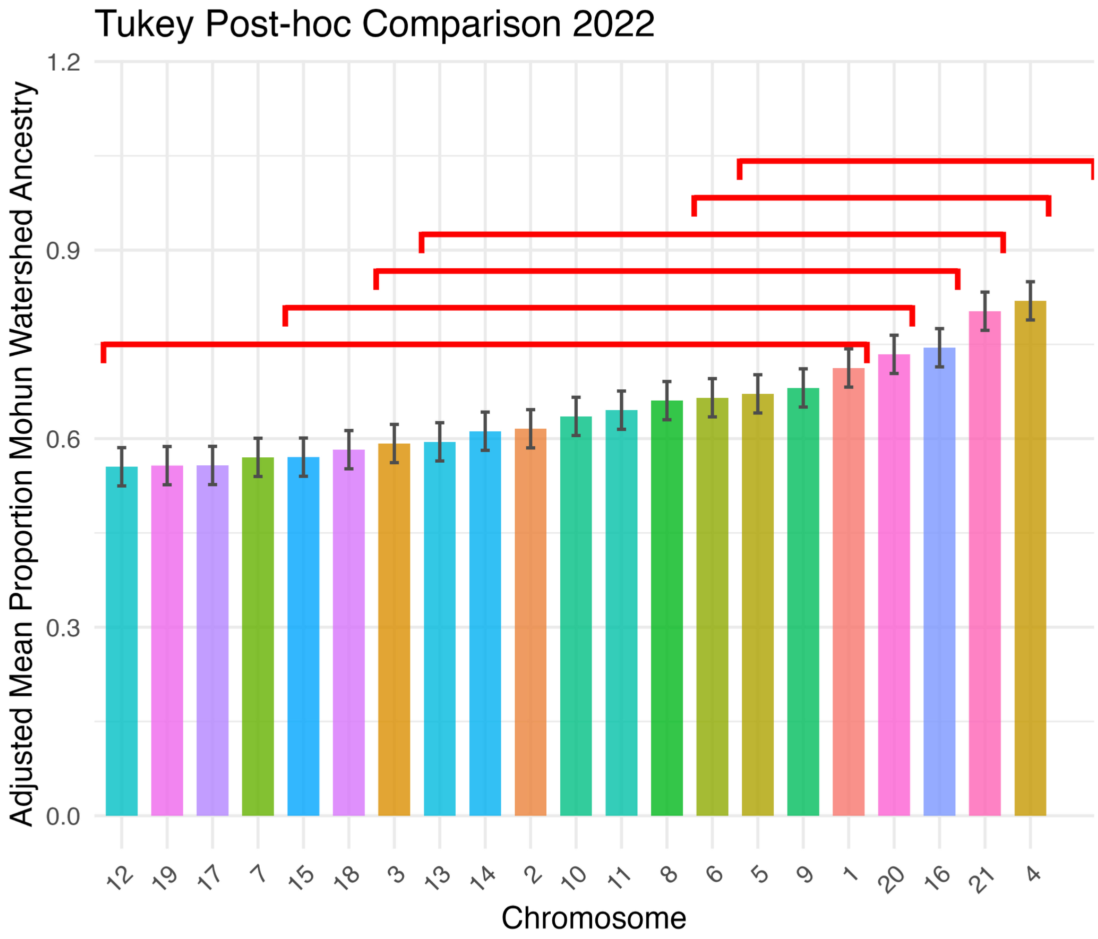

Figure S6: Chromosome-specific differences in Mohun watershed ancestry in 2022 Gosling Lake stickleback. Among-chromosome differences in ancestry proportion in the 2022 sample from Gosling Lake, using an ANOVA to test for overall differences, and Tukey Post-Hoc tests for pairwise differences the red brackets indicate group membership for post-hoc tests. Equivalent tests from 2005-2018 showed no significant differences in ancestry between chromosomes (all P>0.05). The relatively low introgression on the Y chromosome and X chromosome (Chr19) suggest that sexual selection (e.g., inherent fecundity or mate attraction differences) are not driving the introgression documented here.

bioRxiv preprint doi: https://doi.org/10.1101/2025.08.27.672692; this version posted August 28, 2025. The copyright holder for this preprint (which was not certified by peer review) is the author/funder, who has granted bioRxiv a license to display the preprint in perpetuity. It is made available under aCC-BY 4.0 International license.

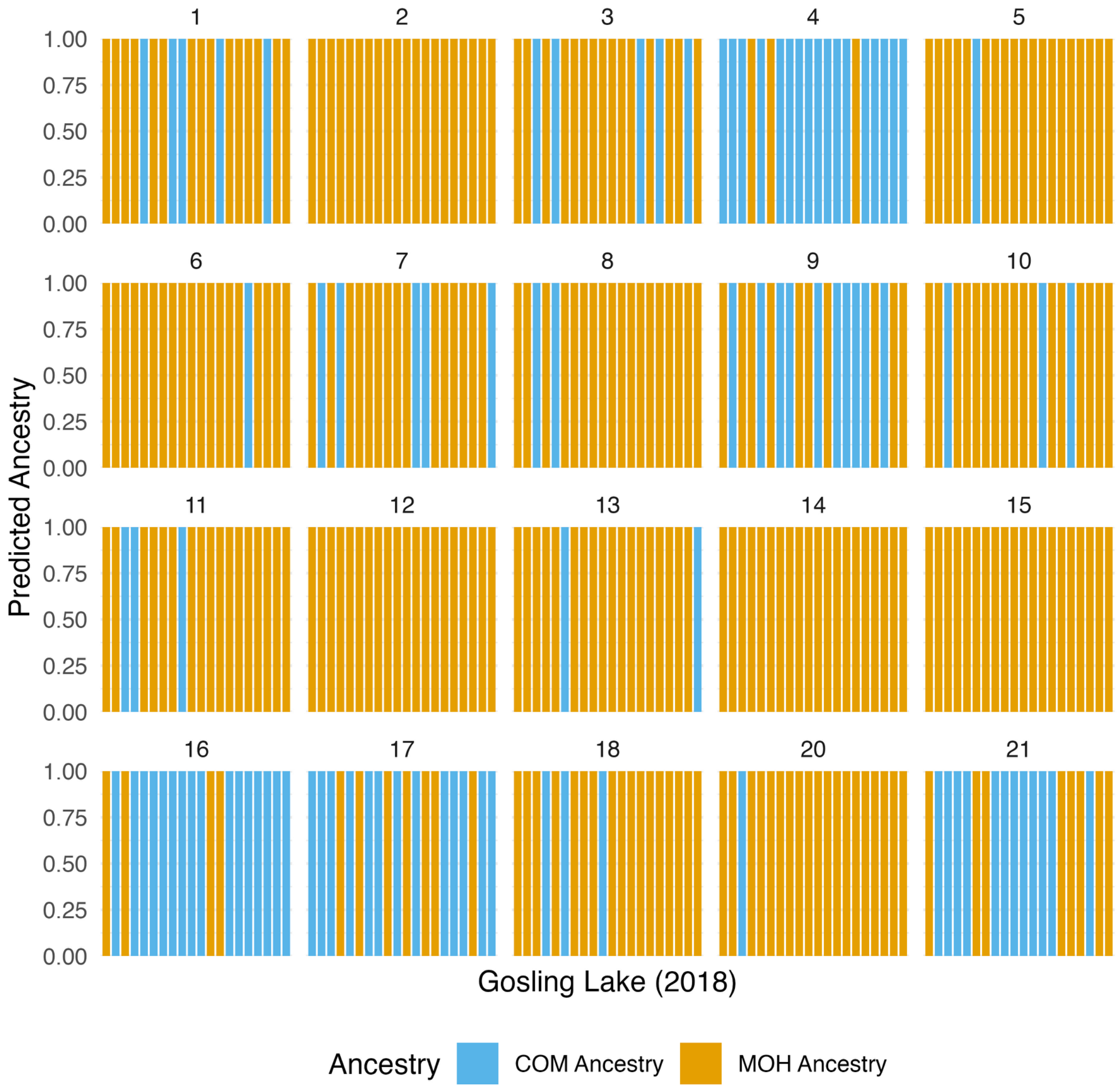

5 **Figure S7: Linear discriminant analysis of principal components.** The predicted population membership of post-invasion Gosling Lake individuals sampled in 2018 to the potential invasion sources from the Mohun watershed (COM/MOH) across all chromosomes. The LDA was trained on a PCA with the hypothesized invasion sources as the grouping variables then used to predict 2018 Gosling Lake individual invasion source membership.

bioRxiv preprint doi: https://doi.org/10.1101/2025.08.27.672692; this version posted August 28, 2025. The copyright holder for this preprint (which was not certified by peer review) is the author/funder, who has granted bioRxiv a license to display the preprint in perpetuity. It is made available under aCC-BY 4.0 International license.

Table S1: Sample sizes by lake and year for low coverage whole-genome sequencing. Lake-year combinations and the number of individual stickleback sampled for whole-genome sequencing at approximately 2X coverage. The 108 individuals sampled from Gosling Lake in 2022 (not shown here) were sequenced at higher depth (~10X coverage).

Sampling Year	Population	Sample Size for low coverage WGS
2023	Boot Lake	19
2023	Comida Lake	20
2009	Comida Lake	19
2007	Gosling Lake	21
2010	Gosling Lake	20
2011	Gosling Lake	20
2012	Gosling Lake	20
2013	Gosling Lake	19
2015	Gosling Lake	20
2018	Gosling Lake	21
2022	Gosling Lake	108*
2009	Higgens Lake	22
2009	Mohun Lake	23
2013	Roberts Lake	22
2009	Sayward Estuary	23

* Used for 10x coverage whole genome sequencing

bioRxiv preprint doi: https://doi.org/10.1101/2025.08.27.672692; this version posted August 28, 2025. The copyright holder for this preprint (which was not certified by peer review) is the author/funder, who has granted bioRxiv a license to display the preprint in perpetuity. It is made available under aCC-BY 4.0 International license.

Tables S2: Genotype counts at the spi1b locus in Gosling Lake from 2005 to 2022. Total number of individuals from Gosling Lake genotyped for the *spi1b* locus by PCR from 2005 to 2022. Genotypes are categorized as *spi1b* -/- (homozygous for the deletion), *spi1b* +/- (heterozygous), and *spi1b* +/+ (homozygous for the full-length allele). PCR genotyping was performed on fin clips using a custom primer set, as described in the Methods.

**Sampling Year**	**Total Genotyped**	***spi1b* -/-**	***spi1b* +/-**	***spi1b* +/+**
2005	30	30	0	0
2007	48	48	0	0
2008	48	48	0	0
2009	38	38	0	0
2010	48	48	0	0
2011	38	38	0	0
2012	48	47	1	0
2013	50	15	30	5
2015	41	7	23	11
2018	12	4	6	2
2022	60	13	24	23

bioRxiv preprint doi: https://doi.org/10.1101/2025.08.27.672692; this version posted August 28, 2025. The copyright holder for this preprint (which was not certified by peer review) is the author/funder, who has granted bioRxiv a license to display the preprint in perpetuity. It is made available under aCC-BY 4.0 International license.

Table S3: Prevalence of S. solidus infections in Gosling Lake stickleback across years. Sample sizes and number of S. solidus infected individuals dissected from Gosling Lake stickleback across multiple years. Years marked with * indicate samples that were dissected from archived formalin preserved fish from Gosling Lake.

Year	N dissected	N infected
2005*	33	14
2006	435	318
2009	147	81
2010	21	10
2012	17	10
2013*	39	15
2014	84	13
2015	60	35
2016	31	13
2022	145	18

bioRxiv preprint doi: https://doi.org/10.1101/2025.08.27.672692; this version posted August 28, 2025. The copyright holder for this preprint (which was not certified by peer review) is the author/funder, who has granted bioRxiv a license to display the preprint in perpetuity. It is made available under aCC-BY 4.0 International license.

Table S4: Frequency of fibrosis in dissected Gosling Lake stickleback across years. Number of Gosling Lake stickleback dissected and recorded as fibrotic across sampling years. Years marked with * indicate samples that were dissected from archived formalin preserved fish from Gosling Lake.

Years	N dissected	N fibrotic
2005*	33	2
2013*	39	20
2014*	33	11
2022	145	6
